# Association Between Nutritional Status, Body Composition, and Disease Activity in Systemic Lupus Erythematosus: An Exploratory Retrospective Study

**DOI:** 10.3390/diseases14070258

**Published:** 2026-07-17

**Authors:** José Luis Sánchez-Reynoso, Sol Ramírez-Ochoa, Berenice Vicente-Hernández, Gabino Cervantes-Guevara, Alejandro González-Ojeda, Clotilde Fuentes-Orozco, Francisco Javier Hernández-Mora, Mauricio Alfredo Ambriz-Alarcón, Luis Asdruval Zepeda-Gutiérrez, Enrique Cervantes-Pérez

**Affiliations:** 1 Departamento de Medicina Interna, Hospital Civil de Guadalajara Fray Antonio Alcalde, Centro Universitario de Ciencias de la Salud, Universidad de Guadalajara, Guadalajara 44280, Jalisco, Mexico; schwarz789@gmail.com (J.L.S.-R.); sramirez@hcg.gob.mx (S.R.-O.); dra.berenicevicente@gmail.com (B.V.-H.); luis-zent@hotmail.com (L.A.Z.-G.); 2 Departamento de Gastroenterología, Hospital Civil de Guadalajara Fray Antonio Alcalde, Guadalajara 44280, Jalisco, Mexico; gabino_guevara@hotmail.com; 3 Departamento de Bienestar y Desarrollo Sustentable, Centro Universitario del Norte, Universidad de Guadalajara, Guadalajara 46200, Jalisco, Mexico; 4 Unidad Biomédica 02, Hospital de Especialidades, Centro Médico Nacional de Occidente, Guadalajara 44350, Jalisco, Mexico; avygail5@gmail.com (A.G.-O.); clotilde.fuentes@gmail.com (C.F.-O.); 5 Departamento de Clínicas de la Reproducción Humana, Crecimiento y Desarrollo Infantil, Centro Universitario de Ciencias de la Salud, Universidad de Guadalajara, Guadalajara 44280, Jalisco, Mexico; frank.gine@gmail.com; 6 Departamento de Obstetricia, Hospital Civil de Guadalajara Fray Antonio Alcalde, Guadalajara 44280, Jalisco, Mexico; 7 División de Medicina, Hospital Civil de Guadalajara Fray Antonio Alcalde, Guadalajara 44280, Jalisco, Mexico; mau_ambriz@hotmail.com; 8 Departamento de Disciplinas Filosóficas, Metodológicas e Instrumentales, Centro Universitario de Ciencias de la Salud, Universidad de Guadalajara, Guadalajara 44340, Jalisco, Mexico; 9 Departamento de Clínicas, Centro Universitario de Tlajomulco, Universidad de Guadalajara, Guadalajara 45641, Jalisco, Mexico

**Keywords:** systemic lupus erythematosus, nutritional status, body composition, disease activity, prognostic nutritional index

## Abstract

**Background:** Nutritional abnormalities, alterations in body composition, and impaired muscle function are increasingly recognized as clinically relevant features in patients with Systemic Lupus Erythematosus (SLE). However, the relationship between multidimensional nutritional assessment and disease activity remains incompletely characterized. This study aimed to evaluate the associations between nutritional status, body composition, muscle strength, nutritional risk indices, and disease activity in patients with SLE. **Materials and Methods:** This exploratory retrospective study included hospitalized patients with SLE. Clinical data, anthropometric measurements, and nutritional status were assessed using the body mass index (BMI), prognostic nutritional index (PNI), controlling nutritional status (CONUT), and nutritional risk index (NRI). Muscle function was evaluated using the SARC-F scale. Disease activity was measured using the SLE Disease Activity Index 2000 (SLEDAI-2K). Multivariable linear regression models were constructed to assess the independent association between nutritional indices and disease activity, adjusting for age, sex, glucocorticoid exposure, and immunosuppressive therapy. Correlation analyses were performed between nutritional indices and disease activity-related variables, and supplementary exploratory Spearman correlations were performed between the SARC-F score and SLEDAI-2K, CRP, complement C3, complement C4, and ESR. False discovery rate correction using the Benjamini–Hochberg procedure was applied to exploratory correlation analyses. Results: A total of 67 patients were included. Nutritional indices (BMI, PNI, CONUT, and NRI) were not independently associated with SLEDAI-2K in multivariable regression analyses. In correlation analyses, after FDR adjustment, the PNI remained significantly associated with SLEDAI-2K, lymphocyte counts, and complement C3 levels, while CONUT remained significantly associated with lymphocyte count and C3 levels. The BMI and NRI were not significantly associated with disease activity or inflammatory markers after correction. SARC-F showed a weak positive correlation with SLEDAI-2K (r = 0.328; *p* = 0.008; qFDR = 0.040), which remained statistically significant after FDR correction. No significant correlations were observed between SARC-F and CRP, complement C3, complement C4, or ESR after FDR adjustment. Conclusions: Nutritional indices showed limited independent association with disease activity in SLE after adjustment for available confounders. Although the PNI and CONUT retained selected correlations with disease activity-related or immunological markers after FDR correction, these associations should be interpreted cautiously, as albumin- and lymphocyte-based indices may reflect inflammatory activity or disease burdens rather than nutritional status alone. SARC-F remains significantly associated with disease activity (SLEDAI-2K) after FDR adjustment. These findings should be validated in future prospective studies with a larger number of patients.

## 1. Introduction

Systemic Lupus Erythematosus (SLE) is a chronic multisystem autoimmune disease characterized by the production of autoantibodies, immune dysregulation, and persistent systemic inflammation with periods of exacerbation and remission [1,2]. Globally, approximately 3.4 million individuals are estimated to live with SLE, with nearly 400,000 new cases diagnosed annually [1]. Despite advances in immunosuppressive therapy and supportive care, patients with SLE continue to experience substantially increased morbidity and mortality compared with the general population, with a marked reduction in life expectancy [3].

Disease activity remains one of the major determinants of adverse outcomes and mortality in SLE. In Latin America, approximately 35% of deaths in patients with SLE have been attributed to disease activity, and in some cohorts, up to 44.9% of deaths have been associated with refractory disease despite immunosuppressive treatment [4]. Several validated indices are currently used to assess disease activity in SLE, including the Systemic Lupus Erythematosus Disease Activity Index 2000 (SLEDAI-2K), the Mexican Systemic Lupus Erythematosus Disease Activity Index (MEX-SLEDAI), and the modified Systemic Lupus Erythematosus Disease Activity Index 2000 (mSLEDAI-2K) [5].

Nutritional abnormalities have increasingly been recognized as clinically relevant factors influencing disease expression, inflammatory burden, physical function, and long-term outcomes in patients with SLE [6,7,8]. Both malnutrition and obesity have been associated with unfavorable clinical outcomes and increased disease activity. Obesity, considered a state of chronic low-grade systemic inflammation, may contribute to increased proinflammatory cytokine production and immune dysregulation [6], whereas malnutrition may impair immune competence and increase susceptibility to disease-related complications [7]. In addition, patients with autoimmune diseases appear to have a higher risk of sarcopenia and impaired muscle function, possibly related to chronic inflammation, reduced physical activity, and corticosteroid exposure, which may further contribute to disability, decreased quality of life, and increased morbidity [9].

The prevalence of nutritional disorders in SLE is considerable. Globally, between 28% and 50% of patients with SLE are estimated to be obese [10], whereas malnutrition has been reported in up to 46.2% of patients in some populations [11]. In Mexico, previous studies have shown that between 50% and 70% of women with SLE are overweight or obese, while approximately 3% are underweight [12,13]. Several studies have explored the relationship between nutritional status and disease activity in SLE, most commonly using body mass index (BMI) as a surrogate marker of nutritional status [6,12,14,15]. However, nutritional assessment is increasingly recognized as a multidimensional construct that extends beyond BMI alone. Nutritional risk indices such as the Prognostic Nutritional Index (PNI), Controlling Nutritional Status (CONUT), and Nutritional Risk Index (NRI), together with body composition analysis and muscle strength assessment, may provide a more comprehensive characterization of nutritional and inflammatory status in patients with SLE [16,17].

Despite growing interest in the interaction between nutritional status and autoimmune disease, there remains limited evidence integrating anthropometric measurements, body composition, muscle strength, nutritional risk indices, and validated disease activity scores in patients with SLE, particularly in Latin American populations. Therefore, this study aimed to evaluate the associations between nutritional status, body composition assessed by bioelectrical impedance analysis, muscle strength assessed by handgrip dynamometry, nutritional risk indices, and disease activity measured using the SLEDAI-2K, MEX-SLEDAI, and mSLEDAI-2K indices in patients with SLE. We hypothesized that multidimensional nutritional assessment parameters would be associated with disease activity and inflammatory burden in this population.

## 2. Materials and Methods

### 2.1. Study Design

This exploratory retrospective study included adult patients with systemic lupus erythematosus (SLE) who were hospitalized in the Internal Medicine Department of Hospital Civil Fray Antonio Alcalde between September 2023 and November 2024. Eligible patients were required to meet all of the following inclusion criteria: (1) age between 18 and 65 years; (2) diagnosis of SLE according to the 2019 European Alliance of Associations for Rheumatology/American College of Rheumatology (EULAR/ACR) classification criteria, which require antinuclear antibody positivity as an entry criterion and a total score of ≥10 points across weighted clinical and immunological domains [18]; and (3) complete clinical, laboratory, anthropometric, bioelectrical impedance, handgrip dynamometry, and nutritional risk index data available in the medical record. Patients were excluded if they were pregnant or breastfeeding, had active malignancy, end-stage renal disease, congestive heart failure, or metallic devices that could interfere with bioelectrical impedance analysis.

### 2.2. Data Collection and Variable Definition

Clinical and laboratory data were retrospectively retrieved from medical records. Laboratory variables included complete blood count, blood chemistry, liver function tests, lipid profile, C-reactive protein, erythrocyte sedimentation rate, and glycated hemoglobin. Immunological parameters included complement C3 and C4 levels, antinuclear antibodies determined by indirect immunofluorescence using HEp-2 substrate, and anti-double-stranded DNA and anti-Smith autoantibodies assessed by immunoassay.

Disease activity was evaluated using the Systemic Lupus Erythematosus Disease Activity Index 2000 (SLEDAI-2K), the Mexican Systemic Lupus Erythematosus Disease Activity Index (MEX-SLEDAI), and the modified Systemic Lupus Erythematosus Disease Activity Index 2000 (mSLEDAI-2K) [5,19].

Anthropometric variables, including weight and height, were obtained and body mass index (BMI) was calculated. Body composition was assessed by segmental multifrequency bioelectrical impedance analysis using the RD-545 InnerScan PRO Body Composition Analyzer (TANITA, Tokyo, Japan). Measurements were performed with patients barefoot and standing according to the manufacturer’s recommendations. The evaluated body composition parameters included the body fat percentage, visceral fat index, bone mass, and skeletal muscle mass index (SMI). Low muscle mass was defined as an SMI <7.0 kg/m^2^ in men and <5.7 kg/m^2^ in women.

Muscle strength was assessed using the EH101 electronic dynamometer (CAMRY, Zhongshan, China). Handgrip strength measurements were performed with the patient seated and the elbow flexed at 90°, using the mean value of three maximal attempts for both dominant and non-dominant hands. Sarcopenia risk screening was performed using the SARC-F questionnaire, in which a score ≥4 points was considered suggestive of sarcopenia [20].

Nutritional risk was assessed using the Prognostic Nutritional Index (PNI), Nutritional Risk Index (NRI), and Controlling Nutritional Status (CONUT) score. These indices were selected because they are objective, reproducible, inexpensive, and can be calculated from routinely available clinical and laboratory variables, which makes them suitable for retrospective studies and clinical settings with variable access to specialized nutritional assessment tools. In addition, the PNI and CONUT incorporate serum albumin and lymphocyte count, variables that may reflect both nutritional reserve and systemic inflammatory or immune status, whereas the NRI combines serum albumin with body weight relative to ideal body weight. Previous studies in patients with SLE, particularly those by Ahn et al. [16,17], have evaluated nutritional indices, including the PNI, CONUT, NRI, and related inflammatory-nutritional markers, in relation to disease activity and inflammatory markers. Therefore, these indices were selected to allow comparison with prior SLE studies and to complement BMI with biochemical and inflammatory components. However, because albumin- and lymphocyte-based indices may be influenced by lupus activity, renal involvement, proteinuria, and systemic inflammation, they were interpreted as composite nutritional–inflammatory indices rather than purely nutritional markers.

The nutritional indices were calculated and classified according to previously validated criteria [21,22,23]. CONUT was calculated as the sum of the serum albumin score, total lymphocyte count score, and total cholesterol score. Serum albumin was scored as follows: ≥3.5 g/dL, 0 points; 3.0–3.49 g/dL, 2 points; 2.5–2.99 g/dL, 4 points; and <2.5 g/dL, 6 points. Total lymphocyte count was scored as follows: ≥1600/mm^3^, 0 points; 1200–1599/mm^3^, 1 point; 800–1199/mm^3^, 2 points; and <800/mm^3^, 3 points. Total cholesterol was scored as follows: ≥180 mg/dL, 0 points; 140–179 mg/dL, 1 point; 100–139 mg/dL, 2 points; and <100 mg/dL, 3 points. The total CONUT score ranges from 0 to 12 and was interpreted as follows: 0–1, normal nutritional status; 2–4, mild malnutrition risk; 5–8, moderate malnutrition risk; and 9–12, severe malnutrition risk [23].

The PNI was calculated using the following formula: PNI = 10 × serum albumin (g/dL) + 0.005 × total lymphocyte count (/mm^3^). The PNI was classified as follows: ≥50, normal; 45 to <50, mild malnutrition risk; 40 to <45, moderate to severe malnutrition risk; and <40, serious malnutrition risk [22].

The NRI was calculated using the following formula: NRI = (1.519 × serum albumin [g/L]) + (41.7 × current body weight [kg]/ideal body weight [kg]). The NRI was classified as follows: >100, no nutritional risk; 97.5–100, mild nutritional risk; 83.5–97.4, moderate nutritional risk; and <83.5, severe nutritional risk [21].

Clinical and laboratory data were compiled into a database using Microsoft Office Excel 2016 (Microsoft Corporation, Redmond, WA, USA). Statistical analyses were performed using SPSS Statistics version 29 (IBM Corp., Armonk, NY, USA).

### 2.3. Statistical Methodology

For descriptive statistics, qualitative variables were expressed as absolute and relative frequencies, whereas quantitative variables were expressed as mean ± standard deviation (SD) for normally distributed data. For inferential analyses of categorical variables, comparisons between groups were performed using the Chi-square test. Patients were stratified according to BMI into three categories: underweight (BMI ≤ 19 kg/m^2^), normal weight (BMI > 19 and <25 kg/m^2^), and overweight/obesity (BMI ≥ 25 kg/m^2^).

The normality of quantitative variables was assessed using the D’Agostino–Pearson and Kolmogorov–Smirnov tests. Comparisons between BMI groups were performed using one-way analysis of variance (ANOVA) for normally distributed continuous variables, followed by Tukey’s post hoc test when appropriate. Correlations between disease activity-related variables and nutritional assessment parameters, including the BMI, PNI, NRI, and CONUT scores, were evaluated using Pearson correlation analysis when distributional assumptions were met. In addition, supplementary exploratory correlation analyses were performed between the SARC-F score and disease activity-related or inflammatory markers, including SLEDAI-2K, C-reactive protein (CRP), complement C3, complement C4, and the erythrocyte sedimentation rate (ESR). Spearman’s rank correlation coefficient was used because of non-parametric distributions. To account for multiple comparisons in exploratory correlation analyses, false discovery rate (FDR) correction using the Benjamini–Hochberg procedure was applied, with FDR-adjusted q values <0.05 considered statistically significant.

Exploratory multivariable linear regression models were constructed to assess whether BMI and nutritional indices (PNI, CONUT, and NRI) were independently associated with disease activity, using SLEDAI-2K as the dependent variable. Separate models were fitted for each nutritional parameter and adjusted for age, sex, glucocorticoid exposure expressed as prednisone-equivalent dose, and current immunosuppressive therapy. No correction for multiple comparisons was applied to the regression models because each model evaluated a distinct prespecified exploratory association. A two-sided *p* value < 0.05 was considered statistically significant for regression analyses.

Albumin was not included as an additional covariate in models evaluating the PNI, CONUT, or NRI because it is a mathematical component of these indices. Including albumin as an independent covariate in the same models could introduce overadjustment and conceptual collinearity.

A two-sided *p*-value < 0.05 with a 95% confidence interval was considered statistically significant in all analyses.

### 2.4. Ethical Statement

The study was conducted in accordance with the Declaration of Helsinki and Good Clinical Practice guidelines. The protocol was reviewed and approved by the Clinical Research and Bioethics Committee of Hospital Civil de Guadalajara Fray Antonio Alcalde (Approval Code: CEI 28/25). Because this was a retrospective study based on previously recorded medical information, with no direct patient contact, no intervention, and no collection of identifiable information for the analysis, the requirement for informed consent was waived by the ethics committee. Data were handled confidentially and analyzed in a de-identified manner.

## 3. Results

A total of 67 patients with SLE were included, of whom 60 (89.6%) were women. The mean age of the cohort was 32.3 ± 12.1 years. The mean BMI was 23.8 ± 6.1 kg/m^2^. According to BMI classification, 52.2% (*n* = 35) of patients had normal weight, whereas 22.4% (*n* = 15) were overweight, 10.5% (*n* = 7) were obese, and 14.9% (*n* = 10) were underweight. The demographic, clinical, disease activity-related, and biochemical characteristics of the study population are summarized in Table 1.

No statistically significant differences in disease activity indices, including SLEDAI-2K, MEX-SLEDAI, and mSLEDAI-2K scores, were observed across BMI categories. Similarly, inflammatory and immunological markers, including the ESR, CRP, complement C3 and C4 levels, leukocyte count, and lymphocyte count, did not significantly differ between BMI groups.

The nutritional assessment, body composition, and physical function results are presented in Table 2. Significant differences between BMI groups were identified for the NRI, body fat percentage, visceral fat index, and bone mass. In contrast, no statistically significant differences were observed for the PNI, CONUT score, SMI, or SARC-F score.

Regarding physical function assessment, 61 patients (92.4%) were classified as having low handgrip strength. In addition, according to the SARC-F questionnaire, 34 patients (50.7%) were considered at high risk for sarcopenia, whereas 33 patients (49.3%) were classified as having low risk for sarcopenia.

Post hoc analyses demonstrated significant differences in body weight, NRI, visceral fat index, and bone mass between underweight and overweight/obesity groups, as well as between normal-weight and overweight/obesity groups. Significant differences in body fat percentage were also identified between underweight and overweight/obesity groups and between normal-weight and overweight/obesity groups (Table 3).

Correlation analyses between nutritional indices and disease activity-related variables are presented in Table 4. After FDR correction, the PNI retained selected correlations with SLEDAI-2K, the lymphocyte count, and complement C3 levels. The PNI showed an inverse correlation with SLEDAI-2K (r = −0.348; *p* = 0.0046; qFDR = 0.0365) and positive correlations with lymphocyte count (r = 0.565; *p* < 0.00001; qFDR = 0.00003) and C3 levels (r = 0.463; *p* = 0.00013; qFDR = 0.00177). The correlations between the PNI and MEX-SLEDAI, mSLEDAI-2K, and C4 were not statistically significant after FDR correction.

CONUT retained significant inverse correlations with the lymphocyte count (r = −0.447; *p* = 0.00019; qFDR = 0.00187) and C3 levels (r = −0.471; *p* = 0.00011; qFDR = 0.00177) after FDR correction. The correlation between CONUT and C4 did not remain statistically significant after correction. The BMI and NRI were not significantly associated with disease activity indices or inflammatory markers after FDR adjustment.

Additional correlation analyses between disease activity indices and body composition or physical function parameters, including body fat percentage, SMI, visceral fat index, bone mass, SARC-F score, and handgrip strength, revealed weak and non-significant correlations (all *p* > 0.05).

Four exploratory multivariable linear regression models were constructed to evaluate whether the BMI, PNI, CONUT, or NRI were independently associated with disease activity measured by SLEDAI-2K after adjustment for age, sex, prednisone-equivalent dose, and current immunosuppressive therapy. The results are shown in Table 5.

Overall, none of the evaluated nutritional indices or BMI showed a statistically significant independent association with SLEDAI-2K after adjustment. CONUT showed a positive but non-significant association with SLEDAI-2K (β = 0.51; *p* = 0.106), whereas the NRI showed a weak inverse but non-significant association (β = −0.04; *p* = 0.275). Similarly, the BMI and PNI were not significantly associated with SLEDAI-2K. Across models, explained variance was low, and no evidence of multicollinearity was observed.

Given the high frequency of sarcopenia risk and reduced handgrip strength observed in this cohort, supplementary exploratory correlation analyses were performed between the SARC-F score and disease activity-related or inflammatory markers, including SLEDAI-2K, CRP, complement C3, complement C4, and the ESR. The results are shown in Table 6.

After FDR adjustment, only the correlation between SARC-F and SLEDAI-2K remained statistically significant (r = 0.328; *p* = 0.008; qFDR = 0.040), whereas all associations with inflammatory biomarkers (CRP, C3, C4, and ESR) were no longer significant.

## 4. Discussion

This study comprehensively evaluated the relationship between nutritional status, body composition, muscle function, and disease activity in hospitalized patients with SLE using anthropometric, biochemical, functional, and nutritional risk assessment tools. Correlation analyses were additionally performed to explore potential associations between nutritional risk indices and validated disease activity scales.

Most previous studies investigating the relationship between nutritional status and disease activity in SLE have primarily focused on BMI [12,15]. More recently, additional nutritional assessment tools, including nutritional risk indices, body composition analysis, and physical performance tests, have been incorporated into clinical research [16,17,24], reflecting the growing recognition that nutritional abnormalities in SLE extend beyond body weight alone.

In the present study, no significant differences in disease activity indices were observed across BMI categories. This finding reinforces the limitations of BMI as an isolated marker of nutritional status in patients with SLE, particularly because BMI does not differentiate between fat mass, lean mass, or body fat distribution. This concept has recently been emphasized in updated approaches to obesity and malnutrition assessment [25]. The mean BMI and the proportion of overweight/obese patients in our study (BMI 23.85 ± 6.09 kg/m^2^; underweight 14.93%, normal weight 52.24%, overweight/obese 32.84%) were lower than those reported by Meza-Meza et al. [12] in Mexican patients with SLE (BMI 27.9 ± 5.34 kg/m^2^; underweight 3.08%, normal weight 26.92%, overweight/obese 70%). In that study, overweight/obese patients presented a higher mean age than the other groups. Our cohort had a lower mean age (32.3 ± 12.12 vs. 40.6 ± 12.6 years), which may partially explain the lower prevalence of overweight and obesity observed in our population.

The underweight group presented the least favorable scores for the PNI, NRI, and CONUT; however, statistical significance was reached only for the NRI scale. This finding may be partially explained by the inclusion of the body weight-to-ideal body weight ratio within the NRI formula, which could predispose underweight patients to lower NRI values compared with overweight or obese individuals.

In our study, no statistically significant differences in disease activity indices (SLEDAI-2K, mSLEDAI-2K, and MEX-SLEDAI) were identified between BMI groups. However, this finding should be interpreted cautiously because the overall sample size was small and some BMI subgroups included a limited number of patients, particularly the underweight and overweight/obesity groups. Therefore, the absence of statistically significant differences across BMI categories should not be interpreted as definitive evidence of no association. These findings contrast with those reported by Meza-Meza et al. [12], who observed higher MEX-SLEDAI scores in overweight or obese patients. Similarly, Kwon et al. [15] reported lower SLEDAI-2K scores in underweight patients compared with non-underweight individuals. The variability observed across studies may reflect the marked clinical, demographic, and therapeutic heterogeneity characteristic of patients with SLE, as prolonged steroid use, immunosuppressive therapy, time since disease diagnosis, and disease severity can independently affect the relationship between BMI and disease activity [14,15].

The disease activity scores observed in our cohort were higher than those reported in previous studies, such as those by Kwon et al. [15] and Ahn et al. [17]. This difference may be related to the characteristics of our study population, as all included patients were hospitalized, whereas previous studies included both outpatient and inpatient populations. Consequently, our results likely reflect a subgroup of patients with greater inflammatory burden and disease severity and should not be generalized to ambulatory patients with mild or moderate disease activity.

Among the evaluated nutritional parameters, the PNI and CONUT retained selected correlations with disease activity-related or immunological markers after FDR correction. These findings are partially consistent with previous studies by Ahn et al. [16,17], which also reported associations between nutritional indices and disease activity in patients with SLE. However, in the present study, none of the evaluated nutritional indices or BMI were independently associated with SLEDAI-2K in exploratory multivariable linear regression models adjusted for age, sex, prednisone-equivalent dose, and current immunosuppressive therapy. Therefore, the observed correlations should be interpreted cautiously and should not be considered evidence of an independent association between nutritional status and disease activity.

The interpretation of the PNI in patients with SLE requires particular caution. Although the PNI is commonly used as a nutritional and prognostic index, its two components, serum albumin and lymphocyte count, are strongly influenced by systemic inflammation and lupus activity. Hypoalbuminemia in SLE may reflect inflammation, renal involvement, proteinuria, or acute-phase response rather than malnutrition alone, whereas lymphopenia is a recognized hematologic manifestation of disease activity and may also be affected by immunosuppressive therapy [26,27,28,29]. Therefore, the observed correlations between the PNI and disease activity or complement levels may indicate inflammatory burden or active disease rather than nutritional status per se. This interpretation is further supported by the multivariable regression analyses, in which the PNI was not independently associated with SLEDAI-2K after adjustment for age, sex, prednisone-equivalent dose, and current immunosuppressive therapy. Because albumin is also included in the calculation of the PNI, CONUT, and NRI, we did not include albumin as an additional covariate in the corresponding multivariable models to avoid overadjustment and conceptual collinearity. This methodological consideration further supports a cautious interpretation of albumin-based nutritional indices in patients with SLE.

Although the disease activity-related markers analyzed in our study are commonly available in routine clinical practice, including in resource-limited settings, several additional immunological markers may provide further insight into inflammatory and disease-activity pathways in SLE. These include anti-DNA antibodies [30], peripheral blood basophils [31], double-negative T cells [32], and complement activation biomarkers, including split products and cell-bound complement activation products [33]. These markers were not included in the present exploratory analysis; therefore, future prospective studies should evaluate whether they improve the characterization of the relationship between nutritional status, inflammatory burden, and disease activity in SLE.

Regarding body composition, overweight/obese patients presented higher body fat percentage and visceral fat index values, as expected. Post hoc analyses demonstrated that these differences were significant primarily between overweight/obese patients and the remaining groups. Significant differences in bone mass were also observed among BMI categories. Since no significant differences in inflammatory markers or disease activity indices were identified between groups, these variations in bone mass may be more closely associated with nutritional and body composition status than with disease activity itself. Previous studies have suggested that obesity-related factors, including increased mechanical loading and hormonal alterations such as elevated estrogen and leptin levels, may positively influence bone mass [34].

Although body composition variables were not consistently associated with disease activity indices, they provided additional characterization of nutritional phenotypes that cannot be captured through BMI alone. The inverse correlation observed between visceral fat and MEX-SLEDAI should be interpreted cautiously, as its biological significance remains uncertain and may reflect residual confounding or disease-related heterogeneity. Additional studies are necessary to clarify the potential interactions between visceral adiposity and disease activity in SLE.

Sarcopenia and muscle dysfunction have increasingly been recognized as clinically relevant manifestations in autoimmune diseases. Previous studies in women with SLE have reported a high prevalence of sarcopenia and reduced handgrip strength compared with healthy controls [24,35]. In our study, 50.74% of patients were classified as having a high probability of sarcopenia according to the SARC-F questionnaire, while 92.42% were classified as having low handgrip strength. These findings suggest that muscle dysfunction may represent an underrecognized clinical burden in hospitalized patients with SLE, although no significant differences were identified between BMI groups.

In our study, SARC-F showed a weak positive correlation with SLEDAI-2K that remained statistically significant after FDR correction (r = 0.328; *p* = 0.008; qFDR = 0.040). However, SARC-F was not significantly correlated with CRP, complement C3, complement C4, or the ESR after FDR adjustment. These findings suggest that patient-reported sarcopenia risk may be related to global disease activity in hospitalized patients with SLE, but not necessarily to individual inflammatory biomarkers in this exploratory analysis, these findings may support the hypothesis that chronic inflammation and glucocorticoid exposure contribute to impaired muscle function in patients with SLE, as previously suggested in autoimmune diseases [24]. However, longitudinal studies are needed to clarify the temporal and causal relationships between disease activity, nutritional abnormalities, and muscle dysfunction.

### Study Limitations

This study has several limitations that should be considered when interpreting the findings. First, the retrospective cross-sectional design precludes any inference regarding temporality, directionality, or causality. Therefore, the observed associations should be interpreted as exploratory and hypothesis-generating rather than causal. Second, the sample size was small, which may have limited statistical power to detect clinically meaningful differences across BMI categories or independent associations in multivariable models. Third, all patients were hospitalized at a single tertiary referral center; therefore, the results may not be generalizable to the broader Mexican SLE population, particularly to ambulatory patients with mild or moderate disease activity. This inpatient-only design may have selected patients with a greater inflammatory burden, higher disease activity, and more severe clinical manifestations. Fourth, important potential confounders, including disease duration, proteinuria, renal involvement, and the presence or absence of lupus nephritis, were not consistently available in the retrospective medical records. Consequently, the prevalence of lupus nephritis, proteinuria, or renal involvement could not be reliably reported, and these variables could not be included in the adjusted models. Because albumin is influenced by renal disease, proteinuria, inflammation, and disease activity, the lack of adjustment for renal involvement is particularly relevant when interpreting associations involving albumin-based indices such as the PNI and NRI. Finally, because all data were obtained retrospectively from medical records, selection bias, measurement variability, and residual confounding cannot be excluded.

Although bioelectrical impedance analysis provides a more comprehensive assessment of nutritional status than BMI alone, it is not considered the gold standard method for body composition analysis, and some of its limitations could potentially be overcome through the use of imaging-based methods such as magnetic resonance imaging or dual-energy X-ray absorptiometry [36,37]. Likewise, the assessment of disease activity partially depends on the availability of biochemical and immunological analyses, which may not always be accessible in resource-limited settings.

Despite these limitations, our study provides a comprehensive multidimensional nutritional assessment in Mexican patients with SLE, integrating anthropometric measurements, body composition analysis, muscle strength evaluation, sarcopenia risk screening, and nutritional risk indices together with validated disease activity scales. To our knowledge, this is one of the first studies in a Mexican population with SLE to incorporate this broad combination of nutritional and functional assessment tools. Collectively, these findings support the incorporation of multidimensional nutritional assessment into the clinical evaluation of patients with SLE and provide preliminary evidence for future prospective studies exploring the relationship between nutritional abnormalities, muscle dysfunction, and disease activity.

## 5. Conclusions

In this exploratory retrospective study of hospitalized patients with SLE, nutritional indices showed limited independent association with disease activity after adjustment for available confounders. Although the PNI and CONUT showed selected correlations with disease activity-related or immunological markers after FDR correction, these associations should be interpreted cautiously. In particular, the PNI may reflect the inflammatory activity or disease burden rather than nutritional status alone because it incorporates serum albumin and lymphocyte counts. BMI was not associated with disease activity indices in the analyses performed, reinforcing the limitations of BMI as an isolated marker of nutritional status in patients with SLE.

Although body composition parameters were not consistently associated with disease activity, the high prevalence of sarcopenia risk and reduced handgrip strength observed in our cohort suggests that functional impairment may represent an important clinical burden in hospitalized patients with SLE. SARC-F showed a weak but statistically significant correlation with SLEDAI-2K after FDR correction; however, this finding should be interpreted cautiously because of the exploratory nature of the analysis and the absence of independent multivariable confirmation. Overall, our findings should be considered hypothesis-generating and warrant confirmation in larger prospective studies specifically designed to evaluate the relationships among nutritional status, muscle dysfunction, and disease activity in SLE.

## Figures and Tables

**Table 1:** Demographic, clinical, disease activity-related, and biochemical characteristics of the study population according to BMI category.

		Total (*n* = 67)		Underweight (*n* = 10)		Normal Weight (*n* = 35)		Overweight/Obese (*n* = 22)		F, (df)	*p*
		Mean	±SD	Mean	±SD	Mean	±SD	Mean	±SD		
Age (years)		32.3	12.12	25	6.89	32.09	12.43	35.95	12.32	2.98, (2)	0.057
Weight (kg)				45.50	4.14	57.57	6.86	77.47	15.99	38.94, (2)	<0.001
				No.	%	No.	%	No.	%		
Gender	Female	60	89.5%	9	90	30	85.70	21	95.50	-	0.504
	Male	7	10.5%	1	10	5	14.30	1	4.50		
Variables related to disease activity											
SLEDAI-2K		11.37	5	16.5	8.5	15.57	7.26	15.55	7.39	0.66, (2)	0.936
MEX-SLEDAI		15.71	7.39	11.3	4.73	11.54	5.19	11.10	5.00	0.05, (2)	0.952
mSLEDAI-2K		14.09	7.74	15.10	9.24	13.97	7.58	13.78	7.59	0.10, (2)	0.904
Platelets (10^9^/L)		191.57	144.23	273.61	133.15	147.47	126.21	224.43	156.24	4.19, (2)	0.190
Leukocytes (10^9^/L)		8.03	5.66	7.86	4.96	7.31	5.58	9.24	6.10	0.78, (2)	0.620
Lymphocytes (10^9^/L)		1.17	0.91	0.92	0.42	1.31	0.92	1.03	1.03	1.06, (2)	0.351
ESR (mm/h)		46.43	28.86	66.00	34.54	46.16	25.22	41.00	31.53	1.78, (2)	0.178
CRP (mg/dL)		6.15	8.82	5.64	6.96	5.95	8.57	6.68	10.21	0.06, (2)	0.938
C3 (mg/dL)		68.91	43.07	62.30	25.07	65.38	43.51	77.04	48.88	0.61, (2)	0.547
C4 (mg/dL)		13.43	18.38	13.20	9.65	13.20	9.65	11.36	11.17	0.51, (2)	0.601
Other biochemical indicators											
Creatinine (mg/dL)		1.43	1.35	1.05	0.81	1.34	1.31	1.73	1.58	1.00, (2)	0.373
Cholesterol (mg/dL)		156	72.71	156.70	94.21	159.37	72.99	150.04	63.79	0.10, (2)	0.900
Triglycerides (mg/dL)		185.68	81.93	191.70	94.96	187.35	76.31	180.09	88.05	0.08, (2)	0.923
Albumin (g/dL)		2.99	0.85	2.76	0.86	3.04	0.86	2.99	0.83	0.40, (2)	0.660
ALT (U/L)		35	40.48	30.70	20.17	36.08	49.78	35.22	30.84	0.67, (2)	0.935
AST (U/L)		78.51	221.43	45.40	32.22	94.80	299.55	67.63	85.35	0.22, (2)	0.79

SD, standard deviation; BMI, body mass index; SLEDAI-2K, Systemic Lupus Erythematosus Disease Activity Index 2000; MEX-SLEDAI, Mexican Systemic Lupus Erythematosus Disease Activity Index; mSLEDAI-2K, modified Systemic Lupus Erythematosus Disease Activity Index 2000; ESR, erythrocyte sedimentation rate; CRP, C-reactive protein; C3, complement C3; C4, complement C4; ALT, alanine aminotransferase; AST, aspartate aminotransferase.

**Table 2:** Nutritional assessment, body composition, and physical function parameters according to BMI category.

		Total (*n* = 67)		Underweight (*n* = 10)		Normal Weight (*n* = 35)		Overweight/Obese (*n* = 22)		F, (df)	*p*
		Mean	±SD	Mean	±SD	Mean	±SD	Mean	±SD		
Nutritional indices											
PNI		35.82	10.22	32.29	9.33	37.19	10.67	35.26	9.85	0.93, (2)	0.399
NRI		111.14	26.6	81.58	10.14	102.71	9.21	137.57	27.38	42.87, (2)	<0.001
CONUT		6.27	3.01	7.40	3.16	5.79	3.00	6.50	2.92	1.19, (2)	0.311
Body composition and physical function											
Body fat (%)		26.46	11.34	17.80	4.88	25.21	11.43	34.70	8.92	8.32, (2)	0.001
SMI		7.4	3.14	5.53	0.83	7.29	3.65	8.68	2.07	2.77, (2)	0.072
Visceral fat index		10.61	5.31	6.94	2.03	9.06	2.86	16.28	6.51	19.54, (2)	<0.001
Bone mass		2.27	0.36	1.92	0.17	2.25	0.35	2.52	0.26	10.47, (2)	<0.001
SARC-F		3.52	2.79	3.20	2.69	3.54	2.85	3.63	2.83	0.08, (2)	0.936
				No.	%	No.	%	No.	%		
Handgrip strength classification	Low handgrip strength	61	92.42	10	100	31	88.60	20	95.20	-	0.407

SD, standard deviation; PNI, Prognostic Nutritional Index; NRI, Nutritional Risk Index; CONUT, Controlling Nutritional Status; SMI, skeletal muscle mass index; SARC-F, Strength, Ambulation, Rising from a chair, Stair climbing, and History of falling questionnaire.

**Table 3:** Post hoc analysis of significant differences between BMI categories.

	Weight	NRI	Body Fat	Visceral Fat	Bone Mass
Underweight vs. Normal weight	0.0062	0.004	0.1364	0.3608	0.0212
Underweight vs. Overweight/Obesity	<0.0001	<0.0001	0.0007	<0.0001	<0.0001
Normal weight vs. Overweight/Obesity	<0.0001	<0.0001	0.0141	<0.0001	0.0216

NRI, Nutritional Risk Index.

**Table 4:** Correlation analysis between nutritional indices and disease activity-related variables in patients with SLE.

	BMI	CONUT	PNI	NRI
	r (*p*; qFDR)	r (*p*; qFDR)	r (*p*; qFDR)	r (*p*; qFDR)
Platelets	−0.082 (0.507; 0.424)	0.076 (0.546; 0.424)	−0.078 (0.534; 0.424)	−0.089 (0.475; 0.424)
Leukocytes	0.082 (0.508; 0.424)	0.089 (0.479; 0.424)	−0.079 (0.527; 0.424)	0.090 (0.472; 0.424)
Lymphocytes	−0.040 (0.750; 0.484)	−0.447 (0.00019; 0.00187)	0.565 (<0.00001; 0.00003)	−0.049 (0.698; 0.484)
CRP	−0.187 (0.159; 0.424)	0.127 (0.349; 0.424)	−0.239 (0.074; 0.246)	−0.155 (0.250; 0.424)
ESR	0.129 (0.297; 0.424)	0.227 (0.069; 0.246)	−0.061 (0.629; 0.484)	0.155 (0.215; 0.424)
C3	0.027 (0.833; 0.424)	−0.471 (0.00011; 0.00177)	0.463 (0.00013; 0.00177)	0.050 (0.698; 0.484)
C4	−0.031 (0.809; 0.424)	−0.284 (0.025; 0.127)	0.269 (0.033; 0.147)	−0.024 (0.851; 0.484)
SLEDAI-2K	−0.106 (0.402; 0.424)	0.237 (0.059; 0.238)	−0.348 (0.0046; 0.0365)	−0.154 (0.223; 0.424)
MEX-SLEDAI	−0.055 (0.665; 0.484)	0.213 (0.092; 0.273)	−0.294 (0.017; 0.099)	−0.111 (0.382; 0.424)
mSLEDAI-2K	−0.106 (0.404; 0.424)	0.212 (0.095; 0.273)	−0.324 (0.0091; 0.060)	−0.156 (0.223; 0.424)

BMI, body mass index; PNI, Prognostic Nutritional Index; CONUT, Controlling Nutritional Status; NRI, Nutritional Risk Index; CRP, C-reactive protein; ESR, erythrocyte sedimentation rate; C3, complement C3; C4, complement C4; SLEDAI-2K, Systemic Lupus Erythematosus Disease Activity Index 2000; MEX-SLEDAI, Mexican Systemic Lupus Erythematosus Disease Activity Index; mSLEDAI-2K, modified Systemic Lupus Erythematosus Disease Activity Index 2000; qFDR, FDR-adjusted q value.

**Table 5:** Exploratory multivariable linear regression models assessing the association between nutritional indices, BMI, and SLEDAI-2K.

Predictor	Unstandardized β	SE	Standardized β	t	*p* Value	95% CI
BMI	0.12	0.15	0.09	0.80	0.427	−0.18 to 0.42
PNI	−0.21	0.18	−0.11	−1.17	0.246	−0.57 to 0.15
CONUT	0.51	0.31	0.21	1.64	0.106	−0.11 to 1.13
NRI	−0.04	0.04	−0.15	−1.10	0.275	−0.12 to 0.03

Each model used SLEDAI-2K as the dependent variable and was adjusted for age, sex, prednisone-equivalent dose, and current immunosuppressive therapy. Separate models were fitted for BMI, PNI, CONUT, and NRI. No significant multicollinearity was observed across models. BMI, body mass index; CI, confidence interval; CONUT, Controlling Nutritional Status; NRI, Nutritional Risk Index; PNI, Prognostic Nutritional Index; SE, standard error; SLEDAI-2K, Systemic Lupus Erythematosus Disease Activity Index 2000.

**Table 6:** Supplementary exploratory correlations between SARC-F score and disease activity-related or inflammatory markers.

Variable	r	*p* Unadjusted	qFDR
SLEDAI-2K	0.328	0.008	0.040
C3	−0.045	0.737	0.809
C4	0.089	0.471	0.809
CRP	0.031	0.809	0.809
ESR	0.085	0.505	0.809

Multiple comparisons were adjusted using the Benjamini–Hochberg false discovery rate (FDR) procedure (n = 5). CRP, C-reactive protein; ESR, erythrocyte sedimentation rate; C3, complement C3; C4, complement C4; SLEDAI-2K, Systemic Lupus Erythematosus Disease Activity Index 2000; qFDR, FDR-adjusted q value.

## Data Availability

The data presented in this study are available on request from the corresponding author due to privacy reasons.

## Abbreviations

SLE	Systemic Lupus Erythematosus
SLEDAI-2K	Systemic Lupus Erythematosus Disease Activity Index 2000
MEX-SLEDAI	Mexican Systemic Lupus Erythematosus Disease Activity Index
mSLEDAI-2K	Modified Systemic Lupus Erythematosus Disease Activity Index
BMI	Body Mass Index
PNI	Prognostic Nutritional Index
CONUT	Controlling Nutritional Status
NRI	Nutritional Risk Index
SMI	Skeletal muscle mass index
SARC-F	Strength, Ambulation, Rising from a chair, Stair climbing, and History of falling questionnaire
SD	Standard deviation
ANOVA	One-way analysis of variance
GCP	Good Clinical Practice
ESR	Erythrocyte sedimentation rate
CRP	C-reactive protein
C3	Complement C3
C4	Complement C4
ALT	Alanine aminotransferase
AST	Aspartate aminotransferase
CI	Confidence interval
MRI	Magnetic Resonance Image
DXA	Dual-energy X-ray Absorptiometry
NLR	Neutrophil-to-Lymphocyte Ratio
FDR	False discovery rate

## References

[1] Siegel C.H., Sammaritano L.R. (2024). Systemic Lupus Erythematosus: A Review. JAMA.

[2] Tsokos G.C., Lo M.S., Costa Reis P., Sullivan K.E. (2016). New insights into the immunopathogenesis of systemic lupus erythematosus. Nat. Rev. Rheumatol..

[3] Zen M., Salmaso L., Barbiellini Amidei C., Fedeli U., Bellio S., Iaccarino L., Giollo A., Doria A., Saia M. (2023). Systemic lupus erythematosus incidence and prevalence in a large population-based study in northeastern Italy. Rheumatology.

[4] Ocampo-Piraquive V., Nieto-Aristizábal I., Cañas C.A., Tobón G.J. (2018). Mortality in systemic lupus erythematosus: Causes, predictors and interventions. Expert Rev. Clin. Immunol..

[5] Uribe A.G., Vilá L.M., McGwin G., Sanchez M.L., Reveille J.D., Alarcón G.S. (2004). The Systemic Lupus Activity Measure-revised, the Mexican Systemic Lupus Erythematosus Disease Activity Index (SLEDAI), and a modified SLEDAI-2K are adequate instruments to measure disease activity in systemic lupus erythematosus. J. Rheumatol..

[6] Oeser A., Chung C.P., Asanuma Y., Avalos I., Stein C.M. (2005). Obesity is an independent contributor to functional capacity and inflammation in systemic lupus erythematosus. Arthritis Rheum..

[7] Correa-Rodríguez M., Pocovi-Gerardino G., Callejas-Rubio J.L., Fernández R.R., Martín-Amada M., Cruz-Caparros M.G., Ortego-Centeno N., Rueda-Medina B. (2019). The Prognostic Nutritional Index and Nutritional Risk Index Are Associated with Disease Activity in Patients with Systemic Lupus Erythematosus. Nutrients.

[8] Pons-Estel G.J., Ugarte-Gil M.F., Alarcón G.S. (2017). Epidemiology of systemic lupus erythematosus. Expert Rev. Clin. Immunol..

[9] An H.J., Tizaoui K., Terrazzino S., Cargnin S., Lee K.H., Nam S.W., Kim J.S., Yang J.W., Lee J.Y., Smith L. (2020). Sarcopenia in Autoimmune and Rheumatic Diseases: A Comprehensive Review. Int. J. Mol. Sci..

[10] Hernández-Negrín H., Ricci M., Mancebo-Sevilla J.J., Sanz-Cánovas J., López-Sampalo A., Cobos-Palacios L., Romero-Gómez C., Pérez de Pedro I., Ayala-Gutiérrez M.D.M., Gómez-Huelgas R. (2022). Obesity, Diabetes, and Cardiovascular Risk Burden in Systemic Lupus Erythematosus: Current Approaches and Knowledge Gaps—A Rapid Scoping Review. Int. J. Environ. Res. Public Health.

[11] Xia L., Yang F., Hayashi N., Ma Y., Yan B., Du Y., Chen S., Xia Y., Feng F., Ma Z. (2024). Investigation of Nutritional Factors and Malnutrition Risk Prediction Model in Hospitalized Patients with Systemic Lupus Erythematosus in China. J. Inflamm. Res..

[12] Meza-Meza M.R., Vizmanos-Lamotte B., Muñoz-Valle J.F., Parra-Rojas I., Garaulet M., Campos-López B., Montoya-Buelna M., Cerpa-Cruz S., Martínez-López E., Oregon-Romero E. (2019). Relationship of Excess Weight with Clinical Activity and Dietary Intake Deficiencies in Systemic Lupus Erythematosus Patients. Nutrients.

[13] Fuentes H.M.N., Andrade O.L., Irazoque P.F. (2016). Síndrome metabólico en mujeres mexicanas con lupus eritematoso sistémico. An. Med. Asoc. Med. Hosp. ABC.

[14] Tedeschi S.K., Barbhaiya M., Malspeis S., Lu B., Sparks J.A., Karlson E.W., Willett W., Costenbader K.H. (2017). Obesity and the risk of systemic lupus erythematosus among women in the Nurses’ Health Studies. Semin. Arthritis Rheum..

[15] Kwon O.C., Park M.C. (2024). Patients with systemic lupus erythematosus who are underweight have distinct disease characteristics. Lupus.

[16] Ahn S.S., Jung S.M., Song J.J., Park Y.B., Lee S.W. (2018). Prognostic nutritional index is correlated with disease activity in patients with systemic lupus erythematosus. Lupus.

[17] Ahn S.S., Yoo J., Jung S.M., Song J.J., Park Y.B., Lee S.W. (2019). Comparison of the Clinical Implications among Five Different Nutritional Indices in Patients with Lupus Nephritis. Nutrients.

[18] Aringer M., Costenbader K., Daikh D., Brinks R., Mosca M., Ramsey-Goldman R., Smolen J.S., Wofsy D., Boumpas D.T., Kamen D.L. (2019). 2019 European League Against Rheumatism/American College of Rheumatology classification criteria for systemic lupus erythematosus. Ann. Rheum. Dis..

[19] Gladman D.D., Ibañez D., Urowitz M.B. (2002). Systemic lupus erythematosus disease activity index 2000. J. Rheumatol..

[20] Bahat G., Erbaş Sacar D. (2021). SARC-F test in sarcopenia and frailty: A narrative review. J. Nutr. Food Process..

[21] Aziz E.F., Javed F., Pratap B., Musat D., Nader A., Pulimi S., Alivar C.L., Herzog E., Kukin M.L. (2011). Malnutrition as assessed by nutritional risk index is associated with worse outcome in patients admitted with acute decompensated heart failure: An ACAP-HF data analysis. Heart Int..

[22] Zhang X., Zhang J., Liu F., Li W., Zhang T., Fang B., Zhang Z., Xie Q., Yang Y., Li X. (2023). Prognostic Nutritional Index (PNI) as a Predictor in Patients with Metabolic Syndrome and Heart Failure. Diabetes Metab. Syndr. Obes..

[23] Ignacio de Ulíbarri J., González-Madroño A., de Villar N.G., González P., González B., Mancha A., Rodríguez F., Fernández G. (2005). CONUT: A tool for controlling nutritional status. First validation in a hospital population. Nutr. Hosp..

[24] Bilici R., Candemir B., Satış H., Alp G.T., Borazan F.Y., Deniz O., Guler A.A., Karadeniz H., Varan H.D., Tufan A. (2024). Frequency of sarcopenia in Turkish women with systemic lupus erythematosus. Lupus Sci. Med..

[25] Rubino F., Cummings D.E., Eckel R.H., Cohen R.V., Wilding J.P.H., Brown W.A., Stanford F.C., Batterham R.L., Farooqi I.S., Farpour-Lambert N.J. (2025). Definition and diagnostic criteria of clinical obesity. Lancet Diabetes Endocrinol..

[26] Kandane-Rathnayake R., Louthrenoo W., Golder V., Luo S.F., Wu Y.J., Lateef A., Cho J., Li Z., An Y., Hamijoyo L. (2021). Independent associations of lymphopenia and neutropenia in patients with systemic lupus erythematosus: A longitudinal, multinational study. Rheumatology.

[27] Vilá L.M., Alarcón G.S., McGwin G., Bastian H.M., Fessler B.J., Reveille J.D. (2006). Systemic lupus erythematosus in a multiethnic US cohort, XXXVII: Association of lymphopenia with clinical manifestations, serologic abnormalities, disease activity, and damage accrual. Arthritis Rheum..

[28] Yip J., Aghdassi E., Su J., Lou W., Reich H., Bargman J., Scholey J., Gladman D.D., Urowitz M.B., Fortin P.R. (2010). Serum albumin as a marker for disease activity in patients with systemic lupus erythematosus. J. Rheumatol..

[29] Gremese E., Bruno D., Varriano V., Perniola S., Petricca L., Ferraccioli G. (2023). Serum Albumin Levels: A Biomarker to Be Repurposed in Different Disease Settings in Clinical Practice. J. Clin. Med..

[30] González Rodríguez C., Aparicio Hernández M., Alarcón Torres I. (2021). Update and clinical management of anti-DNA auto-antibodies. Adv. Lab Med..

[31] Dossybayeva K., Bexeitov Y., Mukusheva Z., Almukhamedova Z., Assylbekova M., Abdukhakimova D., Rakhimzhanova M., Poddighe D. (2022). Analysis of Peripheral Blood Basophils in Pediatric Systemic Lupus Erythematosus. Diagnostics.

[32] Poddighe D., Dossybayeva K., Kozhakhmetov S., Rozenson R., Assylbekova M. (2024). Double-Negative T (DNT) Cells in Patients with Systemic Lupus Erythematosus. Biomedicines.

[33] Ayano M., Horiuchi T. (2023). Complement as a Biomarker for Systemic Lupus Erythematosus. Biomolecules.

[34] Hou J., He C., He W., Yang M., Luo X., Li C. (2020). Obesity and Bone Health: A Complex Link. Front. Cell Dev. Biol..

[35] Sumantri S., Rengganis I., Laksmi P.W., Hidayat R., Koesnoe S., Shatri H. (2021). The impact of low muscle function on health-related quality of life in Indonesian women with systemic lupus erythematosus. Lupus.

[36] Borga M. (2018). MRI adipose tissue and muscle composition analysis-a review of automation techniques. Br. J. Radiol..

[37] Messina C., Albano D., Gitto S., Tofanelli L., Bazzocchi A., Ulivieri F.M., Guglielmi G., Sconfienza L.M. (2020). Body composition with dual energy X-ray absorptiometry: From basics to new tools. Quant. Imaging Med. Surg..

